# Development of prophylactic vaccines against HIV-1

**DOI:** 10.1186/1742-4690-10-72

**Published:** 2013-07-17

**Authors:** Torben Schiffner, Quentin J Sattentau, Lucy Dorrell

**Affiliations:** 1 The Sir William Dunn School of Pathology, The University of Oxford, South Parks Road, Oxford OX1 3RE, UK; 2 The Weatherall Institute of Molecular Medicine, The University of Oxford, The John Radcliffe Hospital, Headington, Oxford OX3 9DS, UK; 3 Oxford NIHR Biomedical Research Centre, NDM Research Building, The University of Oxford, Old Road Campus, Oxford OX3 7FZ, UK

## Abstract

The focus of most current HIV-1 vaccine development is on antibody-based approaches. This is because certain antibody responses correlated with protection from HIV-1 acquisition in the RV144 phase III trial, and because a series of potent and broad spectrum neutralizing antibodies have been isolated from infected individuals. Taken together, these two findings suggest ways forward to develop a neutralizing antibody-based vaccine. However, understanding of the correlates of protection from disease in HIV-1 and other infections strongly suggests that we should not ignore CTL-based research. Here we review recent progress in the field and highlight the challenges implicit in HIV-1 vaccine design and some potential solutions.

## Review

### Introduction

Twenty-five years of research into development of a vaccine to prevent or control HIV-1 infection seems like a long time from the fast-moving perspective of the 21^st^ Century. And despite strong optimism from some areas, we still do not have definitive evidence that a robustly protective vaccine can be made. Nevertheless, during this time we have developed a relatively sophisticated understanding of the two essential elements required for vaccine design: the virus, and the host immune system. The field has jumped from an early start using recombinant soluble antigen based upon the surface envelope glycoprotein (Env) gp120 to elicit antibodies, to a focus on cytotoxic T cell (CTL)-based vaccine design, then back in the past 5 years to an emphasis on antibody-based design [1,2]. This shifting hegemony between the two arms of the adaptive immune response was not until recently underpinned by strong scientific foundations supporting a likelihood of efficacy of one approach over the other. However, progress in two areas has galvanized the HIV-1 vaccine field into an unprecedented sense of purpose and activity. Firstly, the isolation over the past 4 years of a series of monoclonal antibodies (mAb) that potently neutralize a broad spectrum of circulating HIV-1 strains, termed broadly neutralizing mAbs (bNmAb). Their existence testifies to the presence of highly conserved epitopes on the HIV-1 envelope glycoproteins (Env) and the ability of humans to make these responses [3-5]. Secondly, the RV144 phase-III trial that showed significant efficacy (Table 1), and in which reduced risk of infection correlated with certain antibody responses but not with CTL responses [6,7]. This review will discuss this recent progress and highlight the challenges to overcome and strategies underway to develop a prophylactic vaccine, including induction of neutralizing antibodies (NAb) and CTL. It will not deal with either therapeutic vaccination or systems relying on delivery of NAbs by expression from in vivo recombinant vectors.

**Table 1:** Summary of completed phase IIb / III HIV-1 vaccine trials

**Vaccine trial**	**Candidate vaccine(s)**	**Phase**	**N volunteers**	**Intended immune response**	**Result**
VAX 003	Protein: rgp120	III	2500	Antibodies, CD4+ T cells	No efficacy
VAX 004	Protein: rgp120	III	5400	Antibodies, CD4+ T cells	No efficacy
RV144	Pox/protein: ALVAC/rgp120	III	16,403	Antibodies, CD4+ & CD8+ T cells	31% efficacy
HVTN 502/ Merck 023	Adenovirus type 5 (Ad5) gag/pol/nef	IIb	3000	CD8+ & CD4+ T cells	No efficacy
HVTN 503	Ad5 gag/pol/nef	IIb	3000	CD8+ & CD4+ T cells	No efficacy
HVTN 505	DNA-Ad5 gag/pol/nef/env	IIb	2504	Antibodies, CD4+ & CD8+ T cells	No efficacy

### Correlates of protection

The development of a vaccine would be facilitated by knowing what type of immune response is likely to be protective against infection and/or disease [8]. First and foremost, NAbs hold centre stage as effectors of sterilizing immunity against HIV-1. Numerous studies in which bNmAbs have been infused systemically or applied topically to the mucosae of non-human primates (NHP) demonstrate that immunodeficiency virus infection can be completely prevented [9]. Both IgG and IgA are protective at mucous membranes [9,10], and protection can be achieved using relatively modest doses of NAbs that yield circulating levels achievable by active vaccination [11,12]. Combinations of bNmAbs may neutralize close to 100% of circulating viruses in vitro [13,14], and potently supress viraemia in a humanized mouse model in vivo [15]. These results suggest that were such antibody combinations elicited in vivo, this would provide solid protection from infection. Thus the primary aim of the antibody vaccine field is to actively induce bNAbs by immunization. That bNAbs can be elicited by the human B cell repertoire has been extensively demonstrated by the cloning of multiple bNmAbs from HIV-1-infected individuals using novel B cell isolation and cloning techniques [16]. It is important to note that there is some evidence for weak protection of NHP from infection by non-neutralizing antibodies, suggesting that other antibody functions may be relevant [17]. In accord with this, the RV144 clinical trial that showed modest protection against infection revealed that antibodies were the best correlate of reduced risk of infection, despite no evidence for neutralizing activity [7,18]. Thus although the clearest case for protection from infection comes from NAbs, non-neutralizing effector mechanisms should not be ignored.

For CTL-based vaccines, defining the correlates of protection from disease as opposed to infection is more challenging, given the heterogeneity in rate of HIV-1 progression. Long-term control of viral replication is not explained by the magnitude or breadth of CTL responses in most infected individuals but does appear to be correlated with CTL functionality and with targeting of low entropy epitopes that are functionally constrained [19]. Since CTL must recognize viral antigens in the context of host HLA antigens, the new host must by definition become infected. Whether CTL can completely eliminate an established infection is unclear, but robust CTL responses can certainly control viral replication and reduce or abort disease in non-human primate (NHP) models [20,21]. A caveat relating to the potential role of vaccine-elicited CTL in controlling human infection relates to the perceived disconnect between results from NHP models and those from clinical trials. Whereas multiple NHP experiments based on CTL elicitation have demonstrated control of SIV infection, this was not recapitulated in a human efficacy trial, the Merck STEP trial (Table 1) [22]. Thus there remains a question mark with regard to the interpretability of the macaque model for HIV-1 CTL-based vaccine development that will only be resolved with further research and clinical trials [23].

### Antibody-based vaccines

Conventional antiviral vaccines mediate antibody- and/or CTL-based protection, depending largely upon the type of vaccine [8]. Killed and subunit vaccines are poor stimulators of CTL and most likely act principally via antibody-based mechanisms, whereas live-attenuated and vectored vaccines may be potent stimulators of antibodies and CTL. In all cases a robust CD4^+^ T cell response is likely to be required to generate potent effector function and strong immunological memory [8]. Most licensed vaccines are considered to work via antibody-mediated functions [24], and it is therefore satisfying that, as described above, passive antibody transfer studies with a variety of NAbs prevented NHP infection [9,25,26]. The isolation of a number of particularly broad and potent bNmAbs [27-32] is very encouraging because it increases the number of conserved antigenic surfaces on Env that can theoretically be targeted in vaccine design. The challenge now, as has been highlighted in several recent reviews and commentaries [3,33-36], is to turn structural understanding into immunogenicity outcomes. Put another way, we need to understand how to use the epitope of a given bNmAb in a particular antigenic context to elicit the same type of antibody specificity after immunization. For this, a detailed understanding of the structure of Env and the associated epitopes of bNmAbs is essential.

#### Env structure/function analysis and how it informs vaccine design

**Figure 1:**
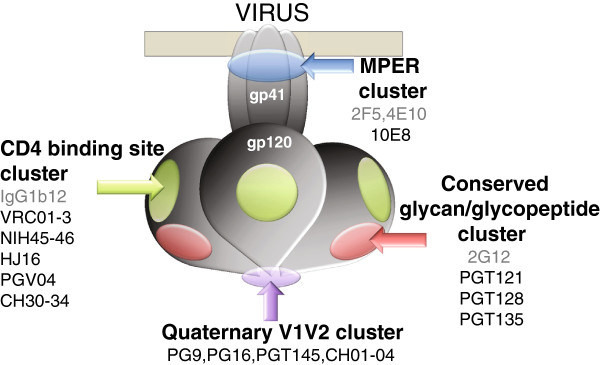
**Model of HIV-1 envelope glycoprotein gross structure and broadly neutralizing antibody binding surfaces.** The functional HIV-1 envelope glycoproteins are made up of two subunits, the outer (surface) receptor binding subunit gp120, and the membrane-spanning, fusion-mediating subunit, gp41. The viral envelope is represented in beige, and the intraviral portion of gp41 is not represented. These subunits are non-covalently linked into a trimer of heterodimers. Glycans are not shown, but there are on average 25 N-linked glycan sites that represent 50% of the total mass of gp120. Broadly neutralizing monoclonal antibody epitope clusters are represented as follows: the gp120 CD4 binding surface (green); an epitope cluster of at the tip of the trimer that depends upon conservation of Env quaternary folding (mauve); the gp120 epitope cluster of glycopeptide-reactive antibodies (pink); the gp41 membrane proximal external region (MPER) cluster (blue). The antibodies listed are non-exhaustive examples of a growing collection. Broadly neutralizing antibodies isolated pre-2009 are lettered in grey, post-2009 in black.

Env is absolutely required by HIV-1 for infection, and is responsible for receptor engagement and virus-cell membrane fusion, functions that are blocked by NAbs. In its mature, functional form Env is comprised of three surface gp120 subunits non-covalently linked to three gp41 transmembrane subunits in the form of a trimer of heterodimers (Figure 1). Since Env is the only virally-encoded antigen accessible to antibodies on the surface of immunodeficiency virus virions and infected cells, vaccine strategies aimed at eliciting protective antibodies must be based on this glycoprotein. However, Env has evolved a number of sophisticated counter-measures to antibody attack that have been elucidated by structure/function analyses. The dominant evasion mechanisms are: rapid amino acid mutation of multiple Env regions [37]; a glycan shield which is to a great extent recognized as ‘self’ by the immune system leading to the minimization of the exposure of underlying conserved protein epitopes [38,39]; steric constraints to Ab binding in the context of individual protomers and of the trimer that protect the CD4bs [40,41] and the CD4-induced CCR5 binding surface [42]; inherent flexibility in the tertiary and quaternary structure of Env called ‘conformational masking’ [43,44]; the presence of immature, misfolded or decayed Env that presents immunodominant non-neutralizing epitopes [45-49]. Understanding of these different evasion mechanisms has greatly informed the field by allowing us to understand which types of surface might make useful vaccine antigens and which, by contrast, are antigenic decoys or vaccine dead-ends.

An important consideration in antibody vaccine design relates to the type of virus that spreads between individuals. In most cases of sexual transmission, infection is established by transfer of a single so-called transmitted/founder (T/F) virus [50-54]. Thus infection presents a bottleneck that might select for T/F viruses with features distinct from the viral swarms that circulate during chronic infection. Analysis of the properties of T/F viruses from different clades has yielded varying conclusions regarding Env structure and function [50-54]. Most studies agree that T/F viruses utilize the co-receptor CCR5, have slightly shorter variable loops and are less glycosylated than chronic strains [50-54]. This under-glycosylation of the T/F viruses might lead to a higher susceptibility to Ab binding, since the glycan shield is a mechanism by which HIV-1 shields conserved epitopes from antibody recognition. However, higher susceptibility of T/F viruses to NmAbs was only found in some studies [50-54]. Thus more research into the properties of T/F viruses is required to inform vaccine design.

Structural information gives insight into the two current major approaches to Env-based vaccine design – that of recapitulating the native trimer in an antigenic form suitable for vaccine use, and that of creating minimalist epitope structures that mimic the surfaces of conserved bNmAb epitopes, so called ‘reverse vaccinology’.

#### How isolation of new bNmAbs changed the field

Given the vast heterogeneity associated with the minimally exposed protein surface of Env, a major question that dominated the field of HIV-1 neutralization for 2 decades was how much Env surface was structurally conserved and Ab accessible. Prototype NmAb isolated in the 1990s recognized three epitope groups on HIV-1 that were conserved between 30-90% of strains; the CD4 binding site (CD4bs) and a glycan epitope on gp120, and the MPER on gp41 (Figure 1). It was unclear whether these NmAbs were examples of extremely rare specificities and therefore very unlikely to be recapitulated by vaccination, or whether these and other bNmAb specificities were relatively common and therefore might provide feasible vaccine targets. The answer is somewhere in between. Between 10-30% (depending on definition of terms and individual cohorts studied) of HIV-1 infected individuals are categorized into what has been termed ‘broad neutralizers’, producing serum Ab responses that neutralize a wide variety of circulating viral strains [55-59]. Although one study suggested that some broad neutralizing responses were the additive result of multiple weak clonal neutralizing responses [60], later work confirmed the existence of at least four independent clusters of highly conserved neutralization epitopes. These are summarized in Figure 1, and incorporate the CD4bs, the MPER, gp120 V1V2 epitopes at the tip of the trimer that are dependent on Env quaternary structure, and a highly glycosylated region at the base of the V3 loop that contains a series of glycan-dependent epitopes. Since these bNmAbs and their characteristics have been the subject of several recent reviews, here we will only summarize the major features of the epitopes that will need to be recapitulated to enable vaccine antigen design.

#### Reductionist approaches to antigen design

A strategy that is being avidly pursued is the design of mimetics based upon atomic-level structural information from bNmAb epitopes. The idea here is to identify the epitope of a bNmAb on the composite antigen, then recapitulate the epitope in a minimal format to focus B cell receptor (BCR) engagement towards that epitope. Because most bNmAb epitopes are conformational and many are discontinuous, this poses a difficult structural problem. However recent progress using *in silico* modelling to design molecular scaffolds to constrain epitopes has resulted in near perfect structural matches being made between the epitope in the context of the original antigen and in its mimetic form. Examples of success in design of such antigenic mimetics are for the MPER 2F5 [61] and 4E10 [62,63] epitopes, and the CD4bs bNmAb 1Gg1b12 [64]. There has also been success in designing Ab-binding surfaces that do not yet have a structurally-defined counterpart in assembled Env or its relevant subunit. Examples are the PG9 [65] and PGT128 [66] bNmAbs, which have been co-crystallized with fragments of gp120 that might make a basis for antigen design, and the MPER-specific bNmAb 10E8 that has a peptide antigen target [67]. Although the complex and unusual nature of many bNmAb epitopes present unprecedented challenges in vaccine antigen design, the large and rapidly growing number of bNmAb-epitope structures encourages optimism that one or more epitopes will be translated into a viable vaccine antigen. However, translating antigenicity in vitro into immunogenicity in vivo is unpredictable and will be a major hurdle to overcome [68-71]. Concerns have been expressed that too much emphasis on basic research towards reductionist structure-based vaccine design will end in frustration and failure [69], but there are equally serious concerns within the vaccine research community that too much emphasis on empirical clinical trials will soak up available funding with an uncertain long-term outcome. Clearly the sensible approach is to do both in parallel, in the reasonable hope that one, or both strategies will pay off in the longer term.

#### Trimer-based antigens

Using the intact Env trimer as a vaccine antigen is a logical approach, since this is the target on the virus (or virus-infected cell) to which NAbs must bind [72]. Moreover, many workers subscribe to the hypothesis that if an Ab can bind with reasonable avidity to a functional Env spike, it will by definition be neutralizing, as occupancy inactivates Env function [72-74]. However, as described above, Env structure is heterogeneous when expressed in a membrane, and preparing soluble forms exacerbates trimer instability and misfolding. Native trimeric antigen may express all bNmAb epitopes, but if they are immunorecessive in the context of the trimer then immunization may fail to elicit the desired responses, or at least at a useful frequency. Moreover, we do not have an atomic-level resolution structure of the complete Env spike – at present cryo-electron microscopy analysis has resolved structures at the molecular level, from 11 - 30 Å [75-79]. Although this allows docking of crystal structure information into a trimer model, details important for immunogen design are lacking, particularly with regard to folding of gp120 variable loops, gp41 structure and the gp120-gp41 interface.

Immunization with currently available soluble forms of HIV-1 Env trimer elicits Ab responses that are only modestly superior to isolated Env fragments such as gp120, and are probably of insufficient potency and breadth of neutralization for vaccine development [80-83]. Thus particular effort is being invested in improving Env trimer homogeneity and stability. Addition of trimerization motifs to the C-terminus of the antigen or elimination of the cleavage site between gp41 and gp120 both improve stability, but fail to direct native folding of the trimer. Addition of targeted inter-protomer disulphide bonds to stabilize the trimer has proven effective, and recent results suggest that so called ‘SOSIP’ trimers from particular viral clones are structurally and antigenically similar to that of native Env [77]. A different and potentially straightforward approach to isolating correctly folded functional Env trimers is based on Env protease resistance. Correctly-folded trimers are proposed to have relative protease resistance compared to misfolded or immature forms, and so can be enriched by exposure to protease [48,84]. Finally, chemical cross-linking may provide stability to the antigen without modifying bNmAb epitope presentation. Aldehyde preserved the binding of a CD4bs bNmAb (IgG1b12) on membrane expressed Env [85], and of a variety of bNmAb epitopes on both soluble and membrane-associated Env forms [44,86,87]. The field is now at the stage of determining whether such antigenic mimics of Env alone can indeed elicit bNmAbs in immunized animals, or whether other additional approaches such as priming with epitope mimetics will be required in addition to select and expand B cells capable of eliciting bNmAbs.

#### Challenges associated with vaccine antigen immunogenicity

The failure to date of Env-based antigens to stimulate bNAb is likely to result from several inter-related reasons that revolve around difficulties in BCR recognition of unusual structural antigenic elements.

1. *Incorrect presentation of the vaccine antigen.* Although the protein component of MPER antibodies can be recapitulated by linear peptides, immunization with linear peptides failed to re-elicit neutralizing responses equivalent to the original mAb [61,63,88-95]. This is in part because the MPER peptide mimics adopted an inappropriate conformation in solution and failed to present the correct surface for B cell recognition. There has been progress in understanding MPER peptide conformation in the context of a lipid environment [96,97], and close mimics of MPER epitopes have now been made [61,63], but the field is held back by the lack of an atomic-level structure of the MPER in the native, non-activated and activation-intermediate forms of gp41.

2. *Cross-reactivity with self.* The 4E10 mAb, and to a lesser extent the 2F5 mAb, bind lipid as part of their epitope by using an array of hydrophobic residues as the tip of their CDR3-like loops. This appears to make them autoreactive [98], and therefore subject to B cell tolerance mechanisms [99]. Although it is unclear whether this is indeed a major barrier in eliciting such mAbs by immunization, the relative rarity of mAbs of this type of specificity would be consistent with this idea.

3. *Epitopes with steric constraints for BCR recognition.* The CD4bs is an obvious target for eliciting NAbs as it requires conservation for function, and needs to be exposed for CD4 binding. Despite this, most infected individuals do not make CD4bs-specific bNmAbs. The principal reason for this appears to result from the intrinsic immunorecessive nature of the conserved segments of CD4bs [70]. As mentioned above, it is physically recessed, allowing ready access of single immunoglobulin domain CD4 but not of the two-domain V region of a BCR [40]. This impediment is aggravated by a second level of steric interference, that of a restricted angle of approach to the target epitope that the BCR must adopt, which is imposed by the oblique angle of presentation of the CD4bs within the intact Env trimer [41] and proximal glycans that reduce Ab access [100,101]. A different example of glycan-imposed steric constraint is found in the ‘glycan canyon’ type epitope, prototype antibodies for which are PG9 and PGT128. These bNmAbs bind epitopes in the V1V2 loop (PG9) and V3 loop base (PGT128), and require an unusually long CDR3 loop to access the peptide ‘floor’ of the canyon whilst also contacting the glycan ‘walls’ [102].

4. *Unique antigenic features for BCR recognition.* The 2G12 bNmAb has an epitope composed entirely of oligomannose groups: epitope mimics prepared so far, despite being immunogenic, have neither elicited Ab with detectable binding to gp120 [103-105] or intact Env trimer [106], nor elicited neutralizing activity after immunization. This is perhaps not surprising given our lack of structural understanding of glycan presentation on Env and the unique architecture of the 2G12 antibody that allows high-affinity glycan recognition required for neutralization [107]. The PG and PGT series of bNmAbs have composite glycan-peptide binding surfaces in which the glycans are heterogeneous [65,66,108]. Preparation of such epitopes will require powerful synthetic chemistry allied to scaffolded peptide design approaches. The MPER bNmAbs 2F5 and 4E10 both require a lipid component to their epitopes [97,109,110] and to date this has not been incorporated into a successful immunogen.

5. *Germline BCR recognition and requirement for extensive antibody affinity maturation.*

There are two probable consequences of the steric constraints imposed on BCRs during recognition of these structurally unusual antigens. The first is that the frequency of germline BCRs available to recognise such complex antigens will be low, therefore a substantial degree of affinity maturation will be required to generate a high-affinity bNAbs able to recognize structurally ‘difficult’ epitopes [111,112]. Alternatively germline BCR affinity for a bNmAb epitope may be undetectable [29,113-115], in which case a different antigenic format may be required to trigger the germline BCR from that required to mature the antibody into its high-affinity bNmAb form [111]. A probable outcome of these constraints is that the host will require long-term antigen exposure to select and clonally expand the rare B cells with appropriate BCRs and to affinity mature them into bNAbs [112], an idea that fits with the observation that most bNmAbs appear to arise in individuals after chronic HIV-1 infection [116].

6. *Conceptual concerns relating to epitope recognition by BCRs*. There are concerns that isolating an epitope from its antigenic context will not lead to re-elicitation of the same type of Ab against the epitope. These concerns stem from the idea that an epitope is a surface defined by a mAb that has undergone a unique process of BCR selection and evolutionary affinity maturation, and due to the stochastic nature of Ab generation and maturation, presentation of the same surface to the immune system will not result in induction of the same unique species of Ab [68,69]. This is a reasonable concern based on proper logic. However it does not take into full account that modern structural biology is able to deconstruct, and reconstruct, the probable pathway a BCR may take in its journey from initial triggering to maturation into a fully functional NAb, both from the point of view of the Ab [111,114,117], and the antigen [117-119]. Moreover, although an epitope mimic may not re-elicit an Ab identical to the template bNmAb, there may be sufficient complementarity between elicited Ab and epitope mimic to allow specific binding to trimeric Env. If this is achieved then trimeric Env may be used to boost and affinity mature those B cells reactive with the epitope mimetic.

7. *Responders and non-responders*. The finding that amongst large cohorts of HIV-1-infected individuals only a minor percentage makes a bNmAb response, suggests that this may apply also to responses to vaccination. Support for the idea that a subpopulation of infected ‘outliers’ may make such responses also comes from a recent NHP study [120]. The questions that this result poses are profound: is bNAb production the consequence of a specific host genetic background, or is it stochastic in that chance favoured the selection and expansion of rare BCRs in only a subset of individuals? Is bNAb elicitation influenced by the ‘type’ of infecting HIV-1 and its evolution within an individual? It will be essential to answer these questions as a priority, as they will to a great extent define how future preclinical NHP and clinical trials are run. If the answer is that genetic background, such as HLA class-II and/or BCR germline are critical bNmAb response determinants, then such potential responders may need to be positively selected or randomized for vaccine trials. If, by contrast, the production of bNAbs is stochastic, then larger groups of individuals will need to be immunized in order to appropriately power the studies.

#### Current strategies and future prospects for development of Ab-based vaccines

Given the list of challenges above it is clear that design of a vaccine to elicit HIV-1 NAbs will not be straightforward, and poses one of the major contemporary challenges to structural biology and immunology. However defining the difficulties is a major step towards solving them. Recapitulating immunorecessive surfaces in isolation from other more immunodominant regions may allow us to overcome the problem of stimulating rare B cell clones. Presentation of an epitope mimic within a variety of different ‘scaffold’ backbones should, in principle, allow focussing of B cell responses to the epitope in question whilst diluting responses to the scaffolds. Although this approach has yet to generate NAbs [61,63], the design, construction and testing of these epitope mimics is still in its infancy, and there are several potential reasons for lack of success. (i) Epitope mimics may not have included all elements for re-elicitation of bNAbs. For example, this is most likely true for the gp41 MPER-specific antibodies 2F5 and 4E10, which require recognition of a lipid component for binding - subsequent designs may strive to make such a component immunogenic. (ii) Animal models used to test constructs may be inadequate. Mice and rabbits are unlikely to be able to recapitulate the same structural features required in a human Ab to effect epitope recognition leading to broad neutralization. For example, they are unlikely to be able to elaborate the long CDR3 loops seen in several bNmAbs [111], and their germline BCR repertoire may fail to engage bNmAb epitopes whereas human germline BCRs may do so. The use of NHPs, humanized mouse models and small phase I clinical trials for testing promising immunogens should overcome this hurdle. (iii) The ability to clonally engage and affinity mature an Ab from germline to mature bNmAb. This may be the most difficult hurdle as it can only be partially overcome by structural biology, and requires manipulation of the immune system to drive the processes intrinsic to Ab production, including BCR triggering leading to clonal expansion and T helper (particularly T follicular helper) cell activation [36,111]. Appropriate immunization models together with new generation adjuvants with defined modes of immune system activation will evolve promising approaches. A requirement for long-term exposure to antigen will drive prolonged immunization schedules and/or antigen expression from persisting vectors. Subsequent small-scale clinical immunogenicity trials will then shed light on which antigen-adjuvant formulations to take forward.

Apart from attempts to induce NmAbs by immunization, further research has gone into the effects of non-neutralizing Ab responses. One such mechanism is antibody-dependent cell-mediated cytotoxicity (ADCC), by which innate immune cells such as NK cells recognize and kill infected cells by detection of Ab bound to viral proteins expressed on the surface of the target cell [121-124]. The significance of Ab effector functions was demonstrated in a study that used passive transfer of the bNmAb b12 to NHPs [11,125]. When challenged with SHIV, the animals were better protected by wild-type Ab than with a mutant that lacked effector functions [11,125]. Further evidence for the importance of Ab effector functions came from follow-up studies of the RV144 clinical trial which found that ADCC correlated with reduced risk of infection in vaccinees that displayed low anti-gp120 IgA titres [7]. It was later shown that high levels of plasma IgA, which does not exhibit ADCC, could block IgG-mediated ADCC in the RV144 patients thus providing a potential explanation for the lack of protection in presence of high IgA levels [126]. Thus antibody-mediated innate immune responses such as ADCC might be part of a protective vaccine, even in the absence of bNmAbs.

### CTL-based vaccines

CD8^+^ T cells play a major role in controlling viral replication during primary immunodeficiency virus infections and in maintaining a stable viral load during the chronic phase. The first effective virus-specific CD8^+^ T cell responses accompany the decline in acute viremia and precede the emergence of NAb by several weeks [127,128]. Experimental depletion of CD8^+^ cells leads to loss of control of SIV replication in infected macaques [129]. HLA class I alleles are a major determinant of viral load set-point and this association is thought to operate through HLA class I-restricted CD8^+^ T cell recognition of susceptible viral epitopes on infected CD4^+^ T cells [130-132]. CD8^+^ T cells drive the selection of escape variants at all stages of disease and early selection of mutant viruses with reduced fitness has been observed in individuals with ‘favourable’ HLA class I alleles [133-136]. NHP models of vaccination against SIV have provided direct evidence to support a T cell-based vaccine approach for HIV-1 infection: although vaccine-induced T cell responses are unable to protect against infectious challenge, they attenuate acute viral replication and viral load set-point in infected animals [20,137-139].

#### What could a CTL vaccine achieve?

By contrast to a NAb response, which aims to prevent acquisition of infection, CTL responses are triggered by recognition of virus-infected host cells. A T cell vaccine could therefore protect against AIDS in one of several ways. If primed T cells are able to home rapidly to mucosal sites they might abort an early focus of infection; however, long-term protection would most likely depend on the maintenance of a high level of fully functional effector cells in the genital tract. A more realistic goal is to attenuate early viral replication and maintain control of viraemia so that disease progression is delayed or avoided and replication is reduced. This would require effective CTL killing, together with a coordinated CD4^+^ T cell helper response. While NHP studies have provided proof of principle, a major stumbling block in translation to humans is that not all T cells are equal. Neither the magnitude nor breadth of HIV-1-specific CTLs correlate with viral load or CD4^+^ cell counts; Gag-specific CD8^+^ T cell responses are strongly associated with HIV-1 control at the population level, however, all viral proteins contain distinct epitopic regions that elicit ‘protective’ and ‘non-protective’ T cell responses [140-142]. Furthermore, the breadth of T cell responses to similar vaccine regimens is considerably greater in macaques than humans [139].

#### Strategies attempted to date

Live-attenuated vaccines generally elicit more potent and durable pathogen-specific immune responses than inactivated or subunit vaccines. However, the development of a live attenuated HIV-1 vaccine is precluded by the inherent risk of vaccine strains acquiring full virulence, as was demonstrated a decade ago with a *nef*-deleted SIV (reviewed in [143]). Delivery of HIV-1 antigens by naked DNA and attenuated viral vectors circumvents this risk, with additional advantages including stability, scope for rational design and capacity for large-scale manufacture. As DNA vaccines are weakly immunogenic when used alone, and viral vector vaccines are rendered ineffective by vector-specific immune responses if used repeatedly, the two approaches have often been tested in combination as prime-boost vaccination strategies. The attenuated poxviruses, canarypox (ALVAC), NYVAC, modified vaccinia Ankara (MVA) have been extensively evaluated as boosting vectors and have an excellent safety record in both healthy and immunocompromised populations (reviewed in [144]). Their capacity to accommodate a large amount of foreign genetic material is an added advantage. However, attenuation comes with the cost of reduced immunogenicity compared to parental virus strains, particularly for induction of CTL. As a consequence, none of the poxvirus-vectored HIV-1 vaccine candidates that have been tested in phase I trials has advanced to phase IIb/III as a pure CTL vaccine concept. Adenoviruses have also been widely tested as vaccine vectors as they are easy to manipulate and manufacture and are highly immunogenic: attenuation is achieved by deletion of the E1 gene, while deletion of additional genes such as E3 enhances immunogenicity further still. Human adenovirus type 5 (Ad5) vectored HIV-1 vaccines were developed independently by Merck and NIH Vaccine Research Centre (VRC). Merck advanced its human Ad5-vectored trivalent HIV-1 clade B *gag/pol/nef* vaccine to early clinical trials on the basis of protective efficacy of DNA prime/Ad5 boost vaccinations against SIV in NHP, which was defined as control of viraemia in vaccinated animals challenged with either pathogenic SIV/HIV (SHIV) or SIVmac [138,145,146]. In phase I trials, Ad5-HIV *gag/pol/nef* and Ad5-HIV *gag* vaccines induced strong and durable HIV-1-specific CD8^+^ and CD4^+^ T cells [147,148]. On a per protein basis, the magnitude of responses was substantially greater than that observed after vaccination with DNA prime/poxvirus boost vaccinations [149,150]. The VRC approach comprised an HIV-1 *gag/pol*/multiclade *env* immunogen vectored by DNA and Ad5. Phase I/II trials were conducted in sites across two continents, enrolling > 1000 volunteers (Table 1). These vaccines were also immunogenic, inducing HIV-1-specific T cell responses in the majority of vaccinees [151-153]. One limitation of both approaches was, however, that the immunogenicity of the Ad5 vaccine component was adversely affected by pre-existing Ad-specific antibodies [147]. As seroprevalence for Ad5 is 60% in Europe and North America and ~90% in sub-Saharan Africa, Ad5-vectored vaccines would be expected to be sub-optimal for deployment in sub-Saharan Africa [154]. The Step trial was therefore designed as a ‘test of concept’ study to determine whether T cell responses induced by the Ad5-HIV-1 *gag/pol/nef* vaccine could prevent infection or control early viraemia post-infection.

#### Step & Phambili trials

The Step and Phambili trials evaluated Merck’s trivalent Ad5-HIV-1 vaccine in high-risk MSM and heterosexual men and women in the Americas and Australia (Step) and heterosexual men and women in South Africa (Phambili, Table 1). The Step trial was prematurely terminated in 2007 on grounds of futility: the vaccine failed to prevent infection or impact on early viraemia, despite inducing T cell responses of similar magnitude and breadth to those observed in earlier trials [22,155]. In addition, the safety of Ad5 as a vaccine vector came under intense scrutiny because of a non-significant trend towards increased risk of HIV-1 infections in vaccinees with pre-existing Ad5-specific NAbs. Other clinical trials involving Ad5-vectored HIV-1 vaccines were consequently suspended, including the Phambili trial and the VRC’s phase IIb HVTN 505 trial. However, post-hoc multivariate analyses of STEP participants indicated that the increase in HIV-1 infections among vaccinees was accounted for largely by men who were uncircumcised and/or had pre-existing Ad5-specific humoral immunity [22]. The VRC phase IIb trial was subsequently scaled down and revised to focus on post-HIV-1 acquisition viraemia and to enrol only circumcised male participants without detectable Ad5 NAbs (http://clinicaltrials.gov/ct2/show/NCT00865566).

In addition to safety concerns, the Step trial exposed some critical limitations in the animal models and in measures of HIV-1-specific immunity that underpinned the vaccine strategy. First, NHP studies employing Ad5-SIV vaccinations followed by SHIV challenge failed to predict the lack of effect of the Ad5-HIV-1 vaccine on viral load set-point post-seroconversion. A low-dose heterologous SIVmac challenge has since been shown to replicate the results of Step more accurately than either SHIV or high-dose SIVmac challenges [156]. Secondly, measures of immunogenicity in phase I/II trials of Ad5-HIV-1 vaccines and in the Step trial proved to be misleading, since vaccinees who acquired HIV-1 infection showed a similar magnitude of response to vaccination to those who did not. Furthermore, the breadth of responses in vaccinees was extremely limited, with CD8^+^ T cell responses targeting a median of one epitope per protein, with a bias towards less conserved epitopes [155,157]. Sieve analysis revealed a vaccine effect on breakthrough HIV-1 sequences, which was consistent with vaccine-induced CTL-mediated selection pressure; however, the significance of these findings is uncertain since measurable CTL responses were weak and had minimal impact on viral load [158,159]. Together, these observations suggest that more potent and broad CD8^+^ T cell responses would be needed to contain early viral replication.

#### Current vector choices

How have lessons from the Step trial influenced the vaccine field? First, while some have questioned the validity of the CTL-based approach, it is widely accepted that coordinated effective B and T cell responses will most likely be needed at mucosal sites in order to prevent or abort infection during the critical window between virus transmission and seeding of local lymphoid tissue. The potency of a vaccine is dependent on several factors that each require consideration, including the vector, the immunogen, adjuvantation and the delivery method. Given the limitations of human Ad5 as a vector, alternatives are being evaluated. These include rare human serotypes such as Ad26 and Ad35 and non-human Ads. Ad26 and Ad35 are not neutralized by Ad5-specific antibodies and have been tested in pre-clinical studies. Ad26 and other rare Ads were less potent than Ad5 when tested for induction of HIV Gag-specific T cells in NHP. However, delivery of an SIV *gag* immunogen using Ad26 prime/Ad5 boost vaccinations conferred partial control of viraemia in macaques challenged with homologous SIVmac [21]. Replication-defective Ad35-vectored HIV-1 vaccines containing *gag*, *pol* and *nef* sequences, with or without *env*, have recently been tested in a phase I clinical trial; this confirmed the safety of the vector but immunogenicity was modest [160]. Chimpanzee Ads (ChAds) that are not cross-neutralized by human Ad-specific antibodies have been developed as vectors for *P. falciparum*, hepatitis C and HIV-1 immunogens [161]. They have proven to be safe and highly immunogenic in phase I clinical trials, inducing transgene-specific T cell responses of >500 IFN-γ SFU/million PBMC after a single priming dose of the recombinant ChAd and >2000 SFU/million PBMC after boosting with MVA expressing the same immunogen [162-164] (http://clinicaltrials.gov/ct2/show/NCT01151319).

The safety and lack of persistence of replication-defective viral vectors is an important consideration for deployment in populations at high risk if acquiring HIV-1, as there is a risk of inadvertent vaccination of people who are already infected. One disadvantage, however, is the short duration of transgene expression, which can lead to rapid waning of responses to the immunogen. Replicating and/or persistent vectors, by contrast, more closely mimic a natural viral infection by inducing potent innate immune responses, which in turn enhance systemic and mucosal adaptive cellular and humoral responses. There is renewed interest in developing replicating vectors for HIV-1 immunogen delivery including adeno-associated virus (AAV), Venezuelan equine encephalitis virus (VEE), vesicular stomatitis virus (VSV) and cytomegalovirus (CMV). Recently, a rhesus CMV-vectored SIV vaccine was shown to induce potent and durable SIV-specific CD8^+^ and CD4^+^ T cell responses that conferred long-term protection against systemic viral infection and CD4^+^ cell depletion in over half of the vaccinated animals that were challenged with pathogenic SIV [20]. Protection was associated with responses of effector memory phenotype and no SIV-specific NAbs were induced by the vaccine. This is the strongest evidence to date that vaccine-induced T cells can confer durable control of an AIDS virus. However, the feasibility of taking this strategy into clinical trials is uncertain, given that the CMV vector could cause disease in the long-term.

The quality of innate immune responses elicited by viral vectors is an important consideration in vaccine design that is now receiving attention. Dendritic cells (DC) capture HIV at mucosal surfaces and activate naïve T cells in draining lymph nodes, therefore, the capacity of viral vectors to target specific DC subsets and the types of signal they induce may be key to eliciting effective adaptive immune responses. Systems biology approaches have been used to interrogate innate and adaptive immunity elicited by yellow fever (YF-17D) and seasonal influenza vaccines. Early gene signatures induced by YF-17D, comprising type 1 interferon, inflammasome and complement genes, were highly predictive of vaccine-specific CD8+ T cell responses [165]. Early molecular signatures also predicted Ab responses to trivalent inactivated influenza vaccine [166]. A systems approach was recently used to define innate immune signatures in recipients of MRK Ad5/HIV vaccine: upregulation of genes associated with inflammation, interferon responses and myeloid cell trafficking was observed in PBMC within 24 hours of vaccination and was accompanied by marked elevations in circulating proinflammatory cytokines [167]. It is noteworthy that these changes were highly attenuated by pre-existing NAbs to Ad5, and the innate response profile predicted the magnitude of subsequent HIV-specific CD8^+^ T cell responses. It is likely that such systems approaches will play an increasingly important role in identifying and selecting vaccine candidates and adjuvants for further development [168].

#### New antigen design

Most HIV-1 vaccine candidates tested to date have incorporated viral gene sequences that were based on consensus or reference sequences representing one or more clades. While full-length structural genes (*gag/pol/env*) have frequently been included, a rational and systematic approach to immunogen design is needed to cope with the enormous global diversity of circulating viral strains. Two novel approaches use isolated conserved regions and mosaics. Engineered genes based on multiple regions that are highly conserved among the major HIV-1 clades have been expressed in various vectors and are now undergoing clinical testing [169] (http://clinicaltrials.gov/ct2/show/NCT01151319). The goal is to focus immune responses on epitopes within which mutational escape from CD8^+^ T cell responses is constrained or disadvantageous. The mosaic approach employs *in silico* algorithms to generate a large number of recombined virus sequences that are then systematically screened for optimum coverage of epitopes in circulating viruses. An HIV-1 *gag/pol/env* mosaic immunogen, when delivered by replication-defective Ad26 vectors to macaques, was shown to induce broader and more cross-reactive T cell responses than vaccines incorporating consensus or single clade sequences [170]. A third approach is to assemble viral sequences based on an unbiased analysis of epitopes targeted by CD8^+^ T cell responses which have been identified as ‘protective’ in large observational studies [142,171,172]. Of note, these latter studies confirmed the importance of *gag* but also identified non-beneficial regions within Gag and beneficial regions in other proteins. It is likely that all these approaches will be further refined once their strengths and weaknesses have been fully determined in clinical trials.

#### Surrogate measures of vaccine efficacy

In the light of negative results of the Step trial, the advancement of new CTL vaccine candidates to efficacy testing will depend on more accurate measures of protective immunity than have been used to date. An important lesson was that the magnitude and breadth of immune responses measured in IFN-γ Elispot and intracellular cytokine assays did not predict the failure of the vaccination strategy. A clear correlate of protection does not exist as no-one has successfully cleared HIV-1 infection by an immune mechanism. There has since been intense scrutiny of ‘natural effective immune control’, indicated by low or undetectable viraemia (‘elite controller’ status) that is observed in <1% of infected populations. Elite controller cohorts are enriched for ‘protective’ HLA class I alleles and show preferential targeting of Gag epitopes by CD8^+^ T cells, over individuals with progressive disease [131,173]. Furthermore, in vitro studies have revealed qualitative differences in HIV-1-specific cellular responses, indicated by superior cytolytic, proliferative, cytokine-producing and antiviral inhibitory capacities [165,174-176]. However, caution is needed in applying these observations to vaccine trials as most studies of elite controllers or long-term nonprogressors have been cross-sectional in design, thus, cause and effect cannot easily be distinguished.

Assessment of viral inhibition by CD8^+^ T cells has recently attracted interest since it provides possibly the most direct measure of antiviral function in vitro. A prospective study of CD8^+^ T cell antiviral function in individuals with recent HIV-1 infection showed that it strongly predicted the rate of CD4^+^ cell decline in the first three years of follow-up and was inversely related to viral load set-point [177]. In addition, CD8^+^ T cell responses induced in HIV-1-uninfected recipients of DNA prime/Ad5 boost HIV-1 vaccine regimens were analyzed in two studies using different viral suppression assays: responses were significantly greater than in placebo recipients but were weaker and more transient than in chronically infected individuals [178,179]. By contrast, high frequencies of IFN-γ producing T cells were detected in some vaccinees. Together, these data suggest that CD8^+^ T cell-mediated viral inhibition in vitro may reflect effective CTL immunity in vivo more closely than cytokine-based assays.

### Unorthodox approaches

It is generally accepted that generating an effector immune response of high titer and quality is the goal of HIV-1 vaccine design. This supposes elicitation of strong CD4^+^ T cell responses to help expand and functionally mature antigen-specific B cells and CTL. Since CTL require a Th1-type biased immune environment to mature, this has been considered desirable attribute of many HIV-1 vaccine approaches. However, a recent study has called this concept into question. Most surprising is the finding that a vaccine based upon inducing tolerance to SIV that elicited no detectable antigen-specific CTL, CD4^+^ T cell or antibody responses, apparently protected all animals from a high dose of SIVmac239 [180]. This immunity was strikingly long-lasting as protection was achieved after 420 days post immunization. Protective responses appeared to be mediated by a novel subset of regulatory CD8^+^ T cells, since their antibody-mediated depletion abolished the protective effect of the vaccine. The authors propose that it is the suppressive effect of the regulatory CD8^+^ T cells on CD4^+^ T cell activation that prevents SIV replication [180]. The protective effect of dampening immune activation is not without precedent, since vaginal treatment of a small group of NHPs with a mildly immunosuppressive agent appeared to render those animals resistant to subsequent intravaginal challenge [181]. Thus the possibility that an anti-inflammatory or even tolerogenic environment might protect from retroviral acquisition is a new possibility that requires confirmation.

## Final conclusions

The question of whether to focus on induction of Ab or CTLs continues to be debated in the HIV-1 field. However, evidence from many other vaccine-preventable infectious diseases indicates that Ab titers correlate with protection from infection, but CTL-mediated immune responses are required for protection against disease [8,24]. This suggests that a dual approach is still warranted. Aspects of CTL vaccine technology such as replicating or persistent vectors may need to be applied to expression of Env-based antigens to allow long-term antigenic exposure in the context of appropriate immune stimulation for bNAb elicitation. Conversely, approaches to elicit bNmAbs may need to be immunologically compatible with the generation of a parallel CTL response. The RV144 trial showed modest protection against infection in low-risk individuals, for which Env V1/V2-specific binding Ab are a possible correlate. The Step trial showed neither protection against infection nor control of initial viraemia despite the presence of vaccine-induced T cell responses, but the responses were limited in breadth and not focused on conserved regions. The results of both trials, while contrary to expectations, have provided much-needed impetus for the development of new approaches and for rigorous re-evaluation of ‘accepted wisdom’. Application of new insights to future vaccine development efforts will be critical to their success.

## Abbreviations

Ad5: Adenovirus type 5; ADCC: Antibody-dependent cell-mediated cytotoxicity; BCR: B cell receptor; bNmAb: Broadly neutralizing monoclonal antibody; CD4bs: CD4 binding site; CTL: Cytotoxic T cell; Env: Envelope glycoprotein; HIV-1: Human immunodeficiency virus-1; mAb: Monoclonal antibody; NAb: Neutralizing antibody; NHP: Non-human primate; NmAb: Neutralizing monoclonal antibody; T/F virus: Transmitted/founder virus.

## Competing interests

The authors declare no competing interests.

## Authors’ contributions

TS, QJS and LD wrote the manuscript. All authors read and approved the final manuscript.
